# Recent Progress of Triboelectric Nanogenerators for Biomedical Sensors: From Design to Application

**DOI:** 10.3390/bios12090697

**Published:** 2022-08-29

**Authors:** Fatemeh Rahimi Sardo, Arash Rayegani, Ali Matin Nazar, Mohammadali Balaghiinaloo, Mohammadhossein Saberian, Syed Agha Hassnain Mohsan, Mohammed H. Alsharif, Ho-Shin Cho

**Affiliations:** 1 Department of Mining Engineering, Shahid Bahonar University of Kerman, Kerman 7616913439, Iran; 2 Department of Civil Engineering, Sharif University of Technology, Azadi Ave, Tehran 1458889694, Iran; 3 Ocean College, Zhejiang University, Zhoushan 316021, China; 4 School of Medicine, Fasa University of Medical Sciences, Fasa 7461686688, Iran; 5 School of Medicine, Zhejiang University, 866 Yuhangtang Rd., Hangzhou 310058, China; 6 Department of Electrical Engineering, College of Electronics and Information Engineering, Sejong University, Seoul 05006, Korea; 7 School of Electronic and Electrical Engineering, Kyungpook National University, Daegu 41566, Korea

**Keywords:** triboelectric nanogenerators (TENG), self-powered sensors, biomedical sensors

## Abstract

Triboelectric nanogenerators (TENG) have gained prominence in recent years, and their structural design is crucial for improvement of energy harvesting performance and sensing. Wearable biosensors can receive information about human health without the need for external charging, with energy instead provided by collection and storage modules that can be integrated into the biosensors. However, the failure to design suitable components for sensing remains a significant challenge associated with biomedical sensors. Therefore, design of TENG structures based on the human body is a considerable challenge, as biomedical sensors, such as implantable and wearable self-powered sensors, have recently advanced. Following a brief introduction of the fundamentals of triboelectric nanogenerators, we describe implantable and wearable self-powered sensors powered by triboelectric nanogenerators. Moreover, we examine the constraints limiting the practical uses of self-powered devices.

## 1. Introduction

**Figure 1:**
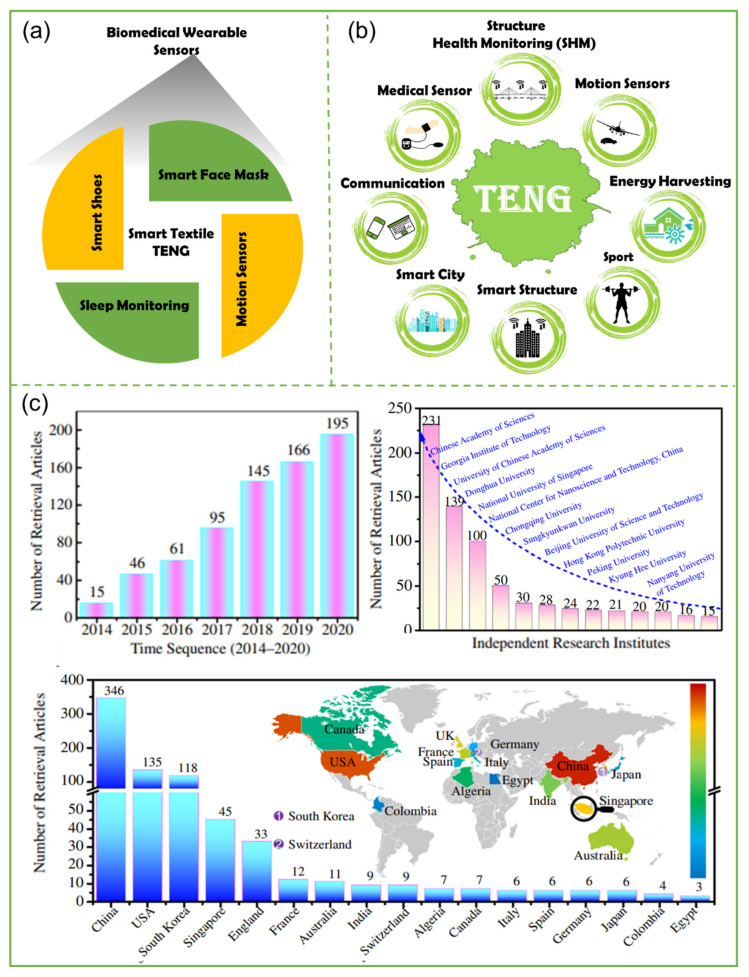
TENG-related publishing trends and applications: (**a**) biomedical wearable sensors based on smart textile TENGs [18]. (**b**) The wide range of applications of TENGs [19]. (**c**) Publications on triboelectric nanogenerator (TENGs) by country [20].

Triboelectric nanogenerators (TENGs) are energy-harvesting devices that use the triboelectric effect to produce electrical power. TENGs are employed in various sectors, including communications (mobile phones), medical (health field) [1,2,3,4], energy absorption from ocean waves [5,6,7,8,9,10,11], and other fields, demonstrating the significant potential of this field in the future. Energy has always been among the most important themes in human history, as is currently the case [12,13,14]. In light of widespread pollution, experts have steadily shifted their focus to clean energy [15]. Triboelectric nanogenerators (TENGs) represent the most significant human discovery in this field in the past decade. Figure 1 shows some of the recent applications of TENGs, such as in medical sensors, motion sensors, and energy harvesting from ocean waves. TENGs offers a wide range of applications beyond energy generation, including in structural health monitoring systems (SHMs) [16,17].

Figure 1a shows the applications of biomedical wearable sensors based on smart textile TENGs [18,19,20]. Several academics shifted their focus to this topic after learning about TENG technology in 2012, and the number of publications published in this field has increased dramatically since then [21,22], representing a significant turning point in the history of mechanical energy harvesting [23]. A variety of TENG applications are depicted in Figure 1b. Researchers have revealed a wide range of uses and potentials for nanogenerators after more than a decade of study in this sector. Whereas TENGs are useful in the production of hydropower and wind energy [8], they can also be used in other fields, such as medicine, civil engineering [18,24] (such as structural health monitoring systems (SHMs) [25] and self-powered sensors [26,27] as an energy source for structures, such as a bridges), and other fields, in order to protect the environment and reduce the production of fossil fuels [28]. An illustration of the distribution of triboelectric nanogenerator (TENG) publications concerning smart textile TENGs for biomedical wearable sensors in various nations is shown in Figure 1c. According to the Scopus database, researchers in several nations are engaged in studying and developing triboelectric nanogenerators (TENGs). Researchers from more than twenty nations have published at least one study on the subject of triboelectric nanogenerators, which should be taken into consideration. According to the statistics provided in Figure 1c, China, the United States of America, South Korea, Singapore, and England are the countries with the most papers published in the area of TENGs (smart textiles), in that order. As research in the field of nanogenerators continues to advance, these publication data can be regarded as optimistic news for a future powered only by clean energy and without pollutants [29].

For sensors to collect, analyze, and transfer data, they require an energy source. Because such sensors may be placed in regions that are not easily accessible to people, the battery may not be able to provide enough power to operate the sensor properly [30,31]. Therefore, researchers are working to eliminate such issues in structural health monitoring systems (SHMs) by including TENGs as a source of energy [32,33,34]. Nations such as the United States of America and China are utilizing TENGs as a source of energy. TENGs have also been adopted as an energy source in the medical engineering field [1,35,36,37,38,39,40,41,42,43], with applications in a variety of medical engineering equipment.

**Figure 2:**
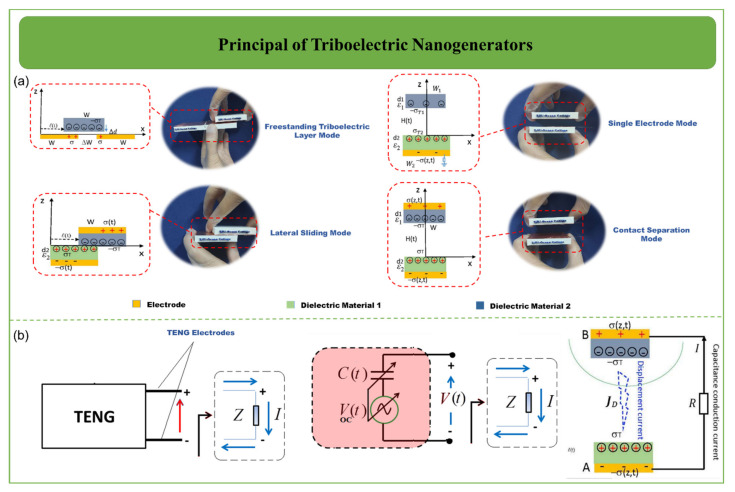
(**a**) The four primary modes of operation for triboelectric nanogenerators include contact separation mode, lateral sliding mode, single-electrode mode, and freestanding triboelectric layer mode. (**b**) Illustration of charge transfer through an external circuit for triboelectric nanogenerators [44,45,46,47].

As shown in Figure 2a, TENGs function in four modes: freestanding triboelectric layer mode, single-electrode mode, lateral sliding mode, and contact separation mode. Each mode has unique properties and advantages. TENG function is based on the transfer of electrostatic charges to the electrodes in each of its modes. All TENG modes use two electrodes, with the exception of the single-electrode mode. When one of the TENG layers is displaced, the electrostatic state is dislodged. This potential difference causes an external charge current. Inverting TENG layers reverses the electrode potential difference. TENG can produce AC using a reciprocating motion. Two electrodes are used in contact separation mode, which is hidden behind the TENG layers. A possible distinction develops during the contact and separation procedures in such a situation [44]. A voltmeter can be used to measure the output voltage by connecting one end to one electrode and the other end to the other electrode. Then, contact and detachment operations can be monitored periodically. Lateral sliding mode comprises two electrodes, which, as in contact separation mode and freestanding triboelectric layer mode, are positioned behind the TENG layers and are referred to as the lateral sliding electrodes. Lateral sliding mode is formed by the relative slide between the TENG layers. The output voltage can be tested using a voltmeter by attaching one end of the voltmeter to one electrode and the other end to the other electrode. The potential voltage difference can be determined by utilizing the TENG layer’s reciprocating slip. Both lateral sliding and contact separation require an electrode output wire, limiting their use. Figure 2b shows charge transfer through an external circuit for triboelectric nanogenerators.

**Figure 3:**
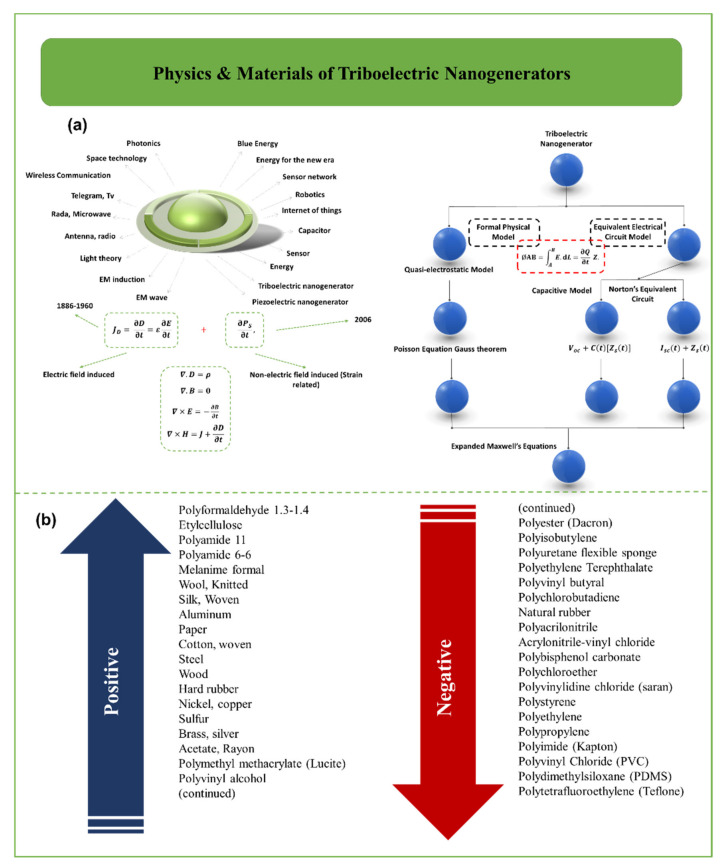
(**a**) Description of the fundamental theories relating to the physics of TENGs. (**b**) Triboelectric series for some common materials [45,64].

As shown in Figure 3a TENG models have been proposed. The first category, which includes the formal physical model [48,49], is executed according to classical electromagnetic theory. 3D mathematical models and the distance-dependent electric field model were developed based on the quasi-electrostatic model [48,50,51,52]. The second category comprises analogous electrical circuit models, including the CA model [53,54,55,56] and the Norton comparable circuit model. The figure shows a transport equation that describes the formal physical model and an analogous electrical circuit model. The two models are linked together. φ_AB_ represents a potential decrease in the TENG system, as expressed on the left side of the equation. Moreover, V = ∂Q/∂t × Z represents the voltage across the external load. According to Kirchhoff’s voltage law, the potential difference between two TENG electrodes is equal to the load resistance voltage. The final product is the transportation equation. The physics of TENGs is determination by the variation of potential (φ), electric field (E), polarization of the dielectric material (P), and the Maxwell’s displacement current (***I***_D_). The circuit models determine the outputs from the external circuit, e.g., variation of voltage (V), current (I), power (P), and extracted electrical energy (E) [50,51]. According to Figure 3a, Maxwell’s equations, known as Wang’s term, are added by the term Ps [45]. Wang’s term is not the result of moderate polarization due to the P electric field but is derived from the existence of electrostatic surface charges:

(1) $$D=\varepsilon_{0}E+P+P_{S}$$

The corresponding displacement current density (***J***_D_) is expressed as:

(2) $$J_{D}=\frac{\partial D}{\partial t}=\varepsilon_{0}\frac{\partial E}{\partial t}+\frac{\partial P}{\partial t}+\frac{\partial P_{S}}{\partial t}=\varepsilon \frac{\partial E}{\partial t}+\frac{\partial P_{S}}{\partial t}$$

where *ɛ*_0_ and *ɛ* are the permittivity of free space (vacuum) and the permittivity of the material (or medium), respectively. These two terms are connected as ɛ_ _≡ ɛ_0_ (1  +  γe), where γe represents the electric susceptibility of the medium. Given that P = (ε − ɛ_0_) E, the volume charge density (Equation (3)) and the density of current density (Equation (4)) are defined by:

(3) $$\rho^{\prime \prime }=\rho -\nabla .P_{S}$$

(4) $$J^{\prime \prime }=J+\frac{\partial P_{S}}{\partial t}$$

Satisfying the charge conversion and continuation equation [45]:

(5) $$\nabla .J^{\prime \prime }+\frac{\partial \rho^{\prime \prime }}{\partial t}=0$$

As a result, Maxwell’s equations are rewritten as [45]:

$$\nabla .D^{\prime \prime }=\rho^{\prime \prime }\;$$

∇.B=0

$$\nabla \times E=-\frac{\partial B}{\partial t}\;$$

(6) $$\nabla \times H=J^{\prime \prime }+\frac{\partial D^{\prime \prime }}{\partial t}$$

The self-consistent equations mentioned above describe the relationships between electromagnetic fields and charges, as well as the current distribution in TENGs [45], where:

(ε∂E/∂t): Well-known contribution to Maxwell’s displacement current; and

(ε∂Ps/∂t): Displacement current due to the presence of surface charges.

(7) $$\varphi \mathrm{AB}=\int_{A}^{B}E.\mathrm{dL}=\frac{\partial Q}{\partial t}Z$$

The equation mentioned in this section is very important, as it serves as a link between the internal circuit and the external circuit. Furthermore, the displacement current (***I***_D_) is obtained by calculating the surface integral (***J***_D_) [44,49,51,57,58,59,60].

(8) $$I_{D}=\int J_{D}.\mathrm{ds}=\int \frac{\partial D}{\partial t}.\mathrm{ds}=\frac{\partial }{\partial t}\int \left(\nabla .D\right)\mathrm{dr}=\frac{\partial }{\partial t}\int \rho \mathrm{dr}=\frac{\partial Q}{\partial t}$$

Figure 3b presents the triboelectric series for some common materials. Triboelectrification phenomena occur in practically commons material, including metals, polymers, silk, and wood, among others [61,62]. As any of these materials can be used to make TENGs, the range of accessible materials for TENG construction is broad. With respect to electron transfer, the capacity of a material to gain or lose electrons is governed by the polarity of the substance. It appears that selecting materials with a wide energy gap results in increased output voltage. Most of the applications discussed this paper involve copper and aluminium as electrode components, with Kapton, PDMS, and PTFE acting as the dielectric material [7,63].

In this review paper, we intend to provide overview and synthesis of the key research developments of TENGs in biological sensors [4,28,37,65,66,67,68,69,70], from design to application. We assessed a wide range of self-powered sensors for human healthcare applications. In Section 4, we review wearable self-powered sensors based on triboelectric nanogenerators [71,72], including smart shoes based on triboelectric nanogenerators, triboelectric nanogenerators for motion sensors, triboelectric nanogenerators for tactile sensors [73], smart face masks based on triboelectric nanogenerators, triboelectric nanogenerators for sleep monitoring, and self-powered nerve/muscle stimulation based on the triboelectric nanogenerators [42]. Finally, in Section 5, we discuss the applications, challenges, and future trends in TENGs for biomedical sensors.

## 2. Overview of Self-Powered Sensors for Human Health Care

**Figure 4:**
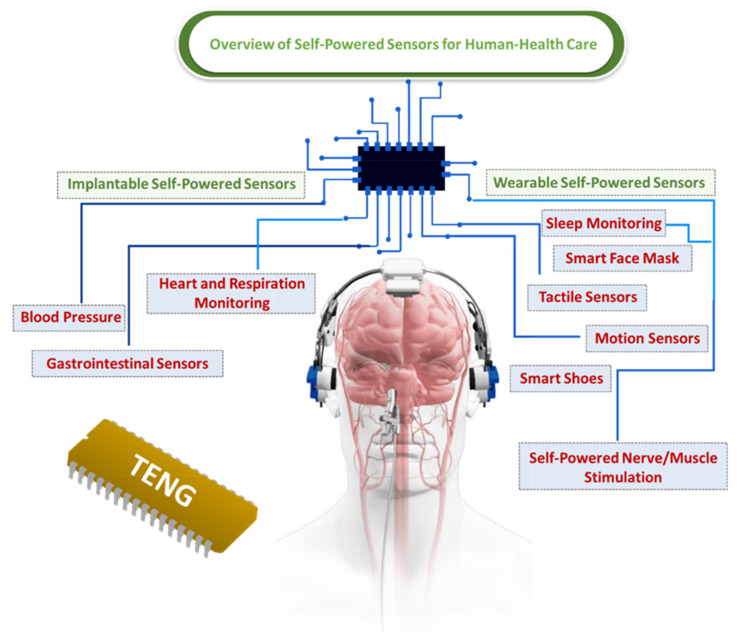
Overview of self-powered sensors for human health care [79].

Wearable biosensors have garnered considerable interest, owing to their ability to provide data that can be used for individualized therapy [40,74]. The majority of molecular biosensors are dependent on electrochemical processes, such as potentiometric, amperometric, differential pulse voltammetric (DPV), and impedance sensing modes. These biosensors can provide information on a molecular level that can be used to indicate human health conditions [75,76]. Such electrochemical sensors provide a high degree of sensitivity and selectivity, in addition to a fast response, and can be easily adapted to wearable devices. The various types of self-powered sensors, including implantable and wearable sensors, are presented in Figure 4 [77,78].

## 3. Implantable Self-Powered Sensors Based on Triboelectric Nanogenerators

Despite the disadvantages of some implantable self-powered sensors, they play key role in medicine science. In the following paragraphs, we will discuss an implanted self-powered sensor that is based on triboelectric nanogenerators and can monitor both the heart respiration, in addition to blood pressure [20,37]. The main applications of such sensors is sensing and monitoring. Most of the applications described in this section involve the use of copper and aluminum as electrode components, with Kapton, PDMS, nylon, silicone rubber, and PTFE acting as the dielectric material.

### 3.1. Triboelectric Nanogenerators for Heart and Respiration Monitoring

**Figure 5:**
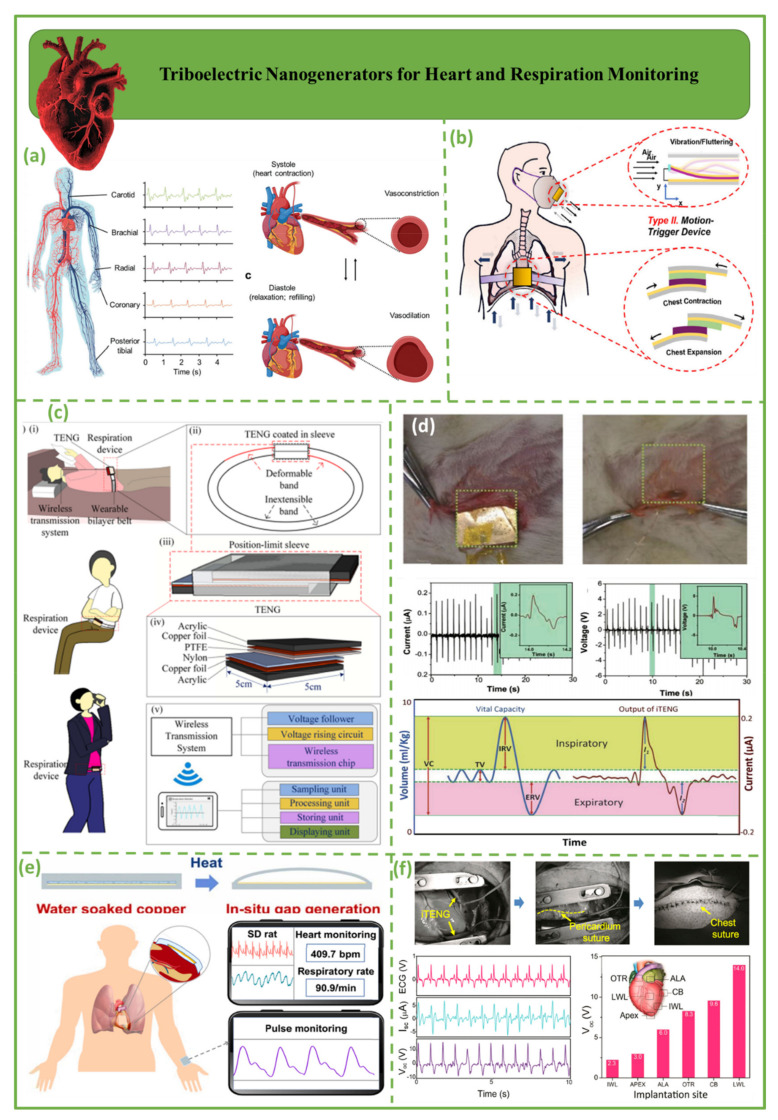
Demonstration of triboelectric nanogenerators for heart and respiration monitoring. (**a**) Wearable triboelectric nanogenerators for heart rate monitoring [81]. (**b**) Respiration-driven triboelectric nanogenerators for biomedical purposes [82]. (**c**) A portable triboelectric nanogenerator to measure respiration in real time [83]. (**d**) An in vivo pacemaker running on an implanted triboelectric nanogenerator powered by the patient’s breathing [87]. (**e**) Eco-friendly in situ gap generation of a no−spacer triboelectric nanogenerator to monitor cardiovascular activities [88]. (**f**) Self−powered wireless cardiac monitoring is provided by the implanted triboelectric nanogenerator in the in vivo system [89].

Figure 5 depicts triboelectric nanogenerators for monitoring the heart and respiration. Figure 5a illustrates sites on the human body that can be monitored with pulse waves derived from actual measurements and biomechanical analysis. In this design, TENG devices are mounted at multiple arterial sites to monitor the heart rate in real time [80,81]. Figure 5b demonstrates biomedical applications of respiration-driven triboelectric nanogenerators. This structure consists of two distinct varieties: type I, for aeroelastic vibration devices; and type II, for motion-triggered devices [82]. Figure 5c shows a wireless respiration sensor that can be worn to track the rate of breathing based on changes in the size of the stomach. The positive and negative tribomaterials are 100 mm PTFE and 30 mm nylon, respectively, and two 50 mm copper foils are affixed to the tribolayers as conducting electrodes. Furthermore, two acrylic sheets provide support for dielectric materials [83]. Figure 5d is an illustration of in vivo biomechanical energy harvesting with a TENG. An implanted triboelectric nanogenerator (iTENG) has also been developed to capture energy from the periodic breathing of a live rat.

The energy produced by breathing was then used to directly operate a prototype pacemaker [84,85,86]. Significant progress has been achieved in the fabrication of TENG-powered implanted medical devices [87]. Figure 5e demonstrates that a wireless mobile system based on NSTENG can identify entire pulse waveforms and display them in real time. This device can detect normal cardiac motion in rats with 99.73 percent accuracy. NSTENG can monitor aberrant cardiac motion and identify minute heart motions that are missed by ECG. Zhao et al. outlined the development of biosafe and novel-structure TENGs, as well as implantable and wearable sensors [88]. Figure 5f shows the iTENG between the heart and pericardium, with the Kapton side facing the left ventricular inferior wall. Periodic cardiac contraction and relaxation triggers the iTENG’s friction layers, causing contact and separation. Experimental resulted indicated a high electrical output, with a Voc of 14 V and an Isc of 5 A in vivo [87].

### 3.2. Triboelectric Nanogenerators for Blood Pressure Sensors

**Figure 6:**
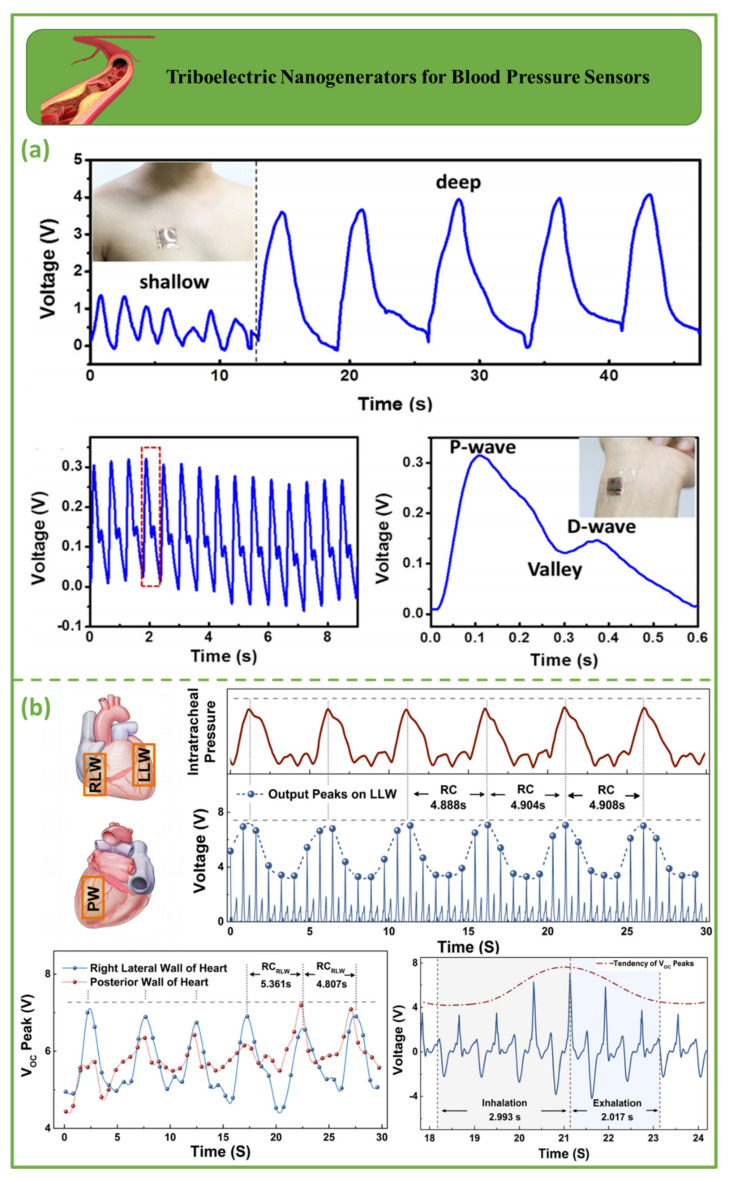
Demonstration of triboelectric nanogenerators for blood pressure sensors. (**a**) Triboelectric nanogenerators based on expandable microspheres as highly sensitive pressure sensors to monitor both the respiratory system and the pulse [91]. (**b**) Schematic representation of the left lateral wall (LLW), right lateral wall (RLW), and posterior wall (PW) from anterior and posterior cardiac views [93].

Blood pressure sensor triboelectric nanogenerators are depicted in Figure 6. Figure 6a illustrates a 33 × 33 mm^2^ chest pressure sensor for respiratory monitoring. In a single breathing cycle, the chest cavity expands and contracts, pushing and releasing the sensor [90]. The 1 min signal indicated shallow and deep breathing modes with 30 and 9 breaths/min, respectively [91]. Figure 6b depicts front and back views of the heart, with three potential implant sites: the left lateral wall (LLW), the right lateral wall (RLW), and the posterior wall (PW) of the heart. When the iTEAS was anchored over the LLW, output voltage peaks fluctuated regularly and steadily. The average time between two consecutive maximum peaks was less than five seconds. The waveforms of the output voltage are indicated by solid blue lines [92]. The blue dots show the maxima of the output voltage. The output peaks of the device placed over the RLW indicate fine but less consistent cyclic variations, whereas the output peaks of the iTEAS positioned under the PW of the heart fluctuated sporadically. During inhalation, output peaks increased from 4.8 to 6.3 V, whereas during cessation, they declined, suggesting separate respiratory phases. The red line represents the inclination of output amplitude fluctuations [93].

## 4. Wearable Self-Powered Sensors Based on Triboelectric Nanogenerators

During the last decade, wearable self-powered sensors based on TENGs have been applied for healthcare monitoring. In this section, we discuss self-powered sensors based on the triboelectric nanogenerators for smart shoes, motion sensors, tactile sensors, smart face masks, sleep monitoring systems, and self-powered nerve/muscle stimulation. Most of the applications discussed in this section involve copper and aluminum as electrode components, with Kapton, PDMS, and PTFE acting as the dielectric material.

### 4.1. Smart Shoes Based on the Triboelectric Nanogenerators

**Figure 7:**
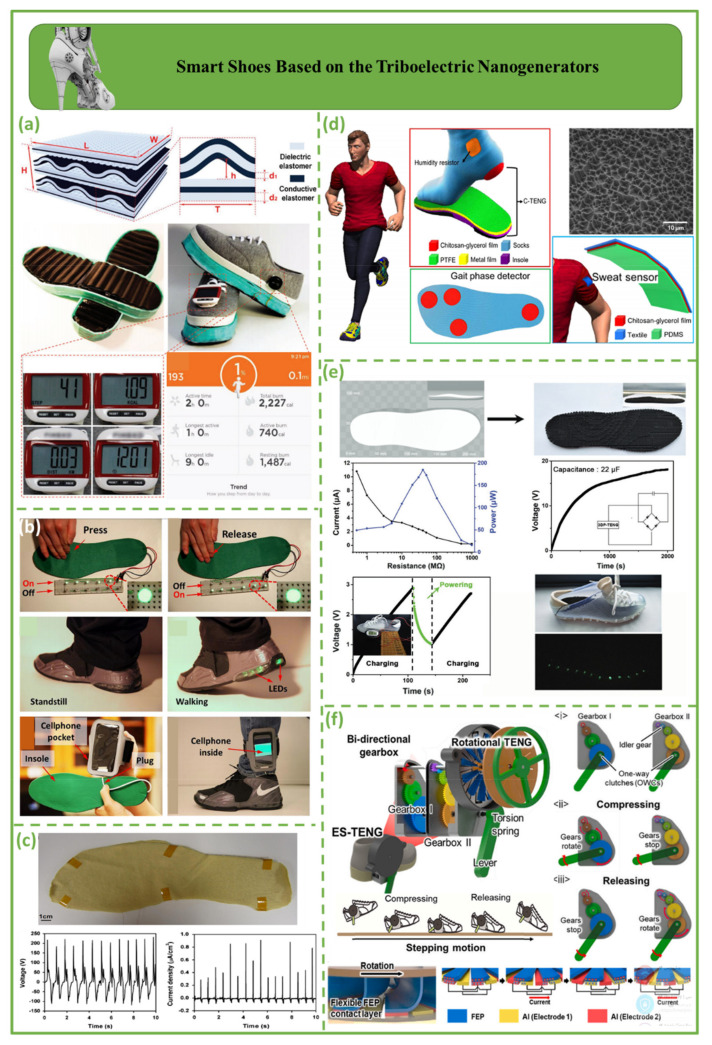
Demonstration of smart shoes based on triboelectric nanogenerators. (**a**) Multilayer elastomeric triboelectric nanogenerators as shoe outsoles [94]. (**b**) Self-lighting shoe with LEDs in the air cushion [95]. (**c**) A TENG−based shoe insole harvests human walking energy to light 30 white LEDs in series [96]. (**d**) Self-powered gait phase detector with four integrated C−TENGs [97]. (**e**) Principle and functionality of 3DP−TENG [98]. (**f**) Schematic illustration of ES−TENG, which consists of a bidirectional gearbox and a rotational TENG [99].

Figure 7 shows smart shoes utilizing triboelectric nanogenerators. Figure 7a demonstrates a self-charging power system using a triboelectric nanogenerator for wearable electronics. The triboelectric nanogenerator is made of multilayered elastomeric arches. A pair of shoes with a maximum equivalent charge current of 16.2 A per shoe comprises a triboelectric nanogenerator on the outsole to gather energy from walking or running [94]. Figure 7b displays a power-generating insole with multilayered triboelectric nanogenerators that record walking pressure. This application is an example of energy-harvesting technology successfully applied for self-powered devices in everyday life, which could have far-reaching ramifications [95].

Figure 7c shows a TENG-based shoe insole that can be used to collect energy from human walking. This particular insole has a maximum output voltage of 220 V and a maximum current density of 40 A. The shoe insole made of a single sheet of TENGs that can be used to illuminate 30 white light-emitting diodes (LEDs) connected in series, which can be achieved by using the TENG sheet as a light source [96]. A successful application of a textile-based, humidity-resistant triboelectric nanogenerator (C-TENG) is shown in Figure 7d. This kind of nanogenerator has the potential to be developed further for self-powered healthcare sensors, for example, for detection of humidity, perspiration, and gait phase. This devices represents a novel application of multifunctional textiles in wearable energy harvesters and self-powered sensors, both of which are promising for the development of future smart apparel items and personalized healthcare sensors [97]. Figure 7e shows that 3D-printed TENGs (3DP-TENGs) can be developed and readily manufactured with a single integrated process that does not require additional assembly stages. The two electrification components of TENGs are poly(glycerol sebacate) (PGS) and carbon nanotubes (CNTs), which also function as electrodes. TENGs are naturally responsive to biomechanical motions owing to their elastic PGS matrix, resulting in robust energy outputs. Chen et al. proposed a novel technique the design and customization of 3D TENGs for varied electronic applications [98]. Figure 7f displays the ES-TENG structure. With efficient motion transformation, the output performance of the proposed ES-TENG is multiplied by more than a factor of ten. Although the ES-TENG does not interrupt the regular stepping motion, daily stepping motion could provide as much as 13 µW/g of specific power. The ES-TENG offers the potential of biomechanical energy harvesting for a wide range of applications by generating considerably improved output performance during natural daily walking motions [99].

### 4.2. Triboelectric Nanogenerators for Motion Sensors

**Figure 8:**
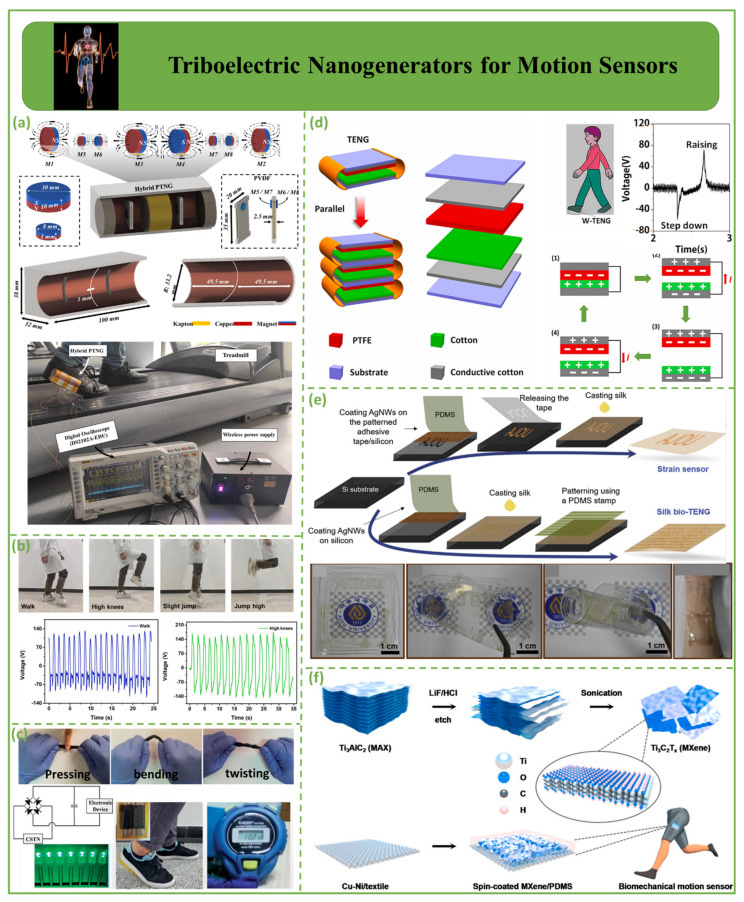
Demonstration of triboelectric nanogenerators for motion sensors. (**a**) PTNG device for energy harvesting and human motion detection [100]. (**b**) SF−TENG monitors various human body motion states [101]. (**c**) A CSTN’s pushing action, bending motion, and twisting motion are demonstrated [102]. (**d**) W−TENG sensor for detection of human walking posture [103]. (**e**) Concept for a silk protein-based strain sensor and triboelectric nanogenerator (TENG) [104]. (**f**) Flexible single-electrode triboelectric nanogenerators for biomechanical motion sensors [105].

Figure 8 shows nanogenerators for triboelectric motion sensors. Figure 8a depicts the development of a PTNG for energy generation and monitoring. In this design, a self-powered walking sensor system leveraging PTNG analyzes human behavior while walking on a treadmill. The highest open-circuit voltage of a hybrid device was measured to be 21.9 V during a treadmill test at varying speeds [100].

Figure 8b depicts a stretchable, highly durable, and deformable triboelectric nanogenerator (F-TENG) based on silica gel that can be used to collect mechanical energy from the environment and monitor human motion and behavior via a highly sensitive response to complex human motion, including stretching, folding, extrusion, and hitting [25,106,107,108]. A study by Zeng et al. promotes the use of TENGs in AI, sports monitoring, and large-scale data collection [101]. Figure 8c shows a flexible, lightweight, biocompatible, coaxially structured triboelectric nanogenerator (CSTN). The device is wrapped in silicone rubber, with an interior hollow circular tube as the core and outside hollow circular tubes as the shell to prevent environmental contamination [109,110]. CSTNs can produce various electric signals at different deformation angles. Therefore, a simple angle sensor function can be implemented in self-powered lighting devices [111], portable electronics, motion detection, and health monitoring [102]. As shown in Figure 8d, cotton film was used as the triboelectric material in the construction of a unique wearable TENG (W-TENG). Triboelectric materials include polytetrafluoroethylene (PTFE) and cotton films. W-TENGs can be used to collect low-frequency mechanical energy from the environment, notably mechanical energy from the human body, and convert it to electrical energy [112]. Furthermore, W-TENGs can be used as human motion sensors to detect a person’s walking position [103].

Figure 8e shows nanostructured silk protein and silver nanowires (AgNWs) embedded in nanostructured silk to generate a skin/textile-compatible, efficient, flexible, transparent triboelectric nanogenerator (TENG) and strain sensor for biomechanical energy harvesting and motion sensing. Strain sensors and bio-TENGs are combined on a single silk chip to sense strain and absorb biomechanical energy [104]. The inexpensive, simple, biocompatible, flexible, and transparent protein-based energy skin offers several advantages [113,114]. Figure 8f illustrates a flexible, single-electrode MXene/polydimethylsiloxane nanogenerator. High output provides 80 green LEDs in sequence with no extra electricity. The textile-based composite can detect finger tapping, hand clapping, and hand hammering, in addition to functioning as a triboelectric nanogenerator. These smart materials can serve as energy sources [105].

### 4.3. Triboelectric Nanogenerators for Tactile Sensors

**Figure 9:**
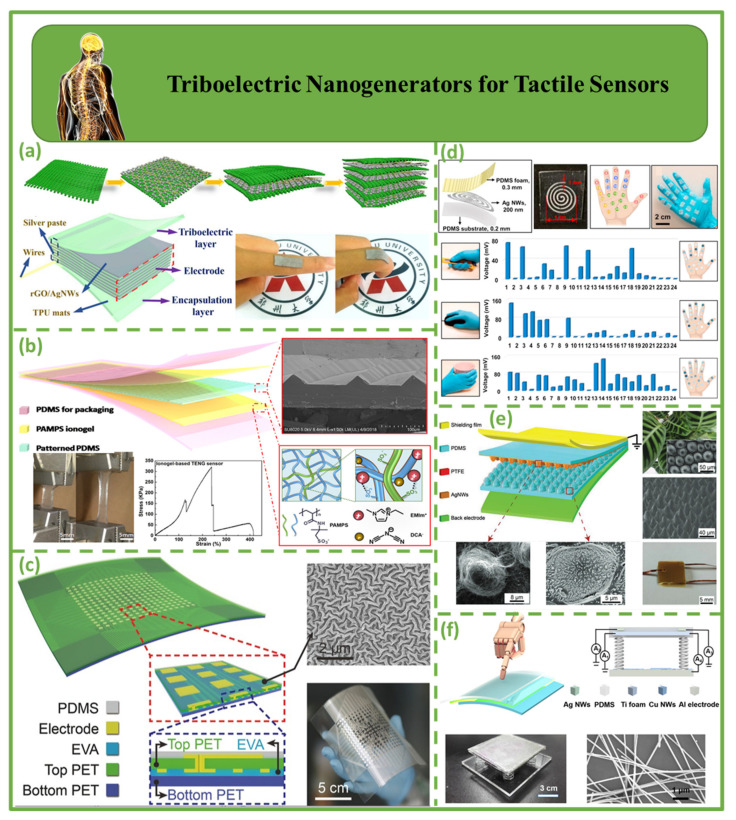
Demonstration of triboelectric nanogenerators for tactile sensors. (**a**) Structural diagram of STENG-based e-skin [115]. (**b**) Transparent and stretchy TENG-based tactile sensor structure [116]. (**c**) TESM structure for real-time tactile mapping [78]. (**d**) Foam-based TENGs as tactile glove sensors [119]. (**e**) Diagrammatic representation of the mechanism of TENG e-skin sensors [120]. (**f**) Principle of smart tactile sensors [121].

Figure 9 depicts nanogenerators for triboelectric tactile sensors. Figure 9a demonstrates a self-powered and flexible electronic skin (e-skin) based on an ultra-stretchable triboelectric nanogenerator (STENG) employing thermoplastic polyurethane/silver nanowires/reduced graphene oxide (rGO). The e-skin (2 × 2 cm^2^) has a high open-circuit voltage (202.4 V) and instantaneous power density (6 mW/m^2^). Zhou et al. proposed a unique and implementable approach for building self-powered, high-performance e-skin for soft robotics, HMI, and the IoT [115]. Figure 9b shows transparent and flexible triboelectric nanogenerators based on ionogel and polydimethylsiloxane film for tactile sensing. Such nanogenerators can monitor physiological processes with sensitivity at varied tensile ratios [116]. Figure 9c shows a single-electrode, self-powered triboelectric sensor matrix. As the flexible dielectric substrate, a 250 m thick PET film square was used to coat with masks with unique patterns on both sides, and laser-drilled through-holes were assembled [109,117]. Magnetron sputtering was used to deposit patterned Ag electrodes on both sides of the substrate once the mask was removed [118]. This device could be used for motion tracking, touch sensing [65], and human–machine interactions [78]. Ultra-thin, soft, skin-integrated, self-powered sensors based on FS-TENGs with porous poly(dimethylsiloxane) foam and sophisticated serpentine silver nanowires are designed for applications of TENGs in sensitive human–machine interactions (Figure 9d). FS-TENGs with 24 integrated sensors on a glove and a tactile sensor array exhibit self-powered sensing and energy harvesting [119]. Figure 9e shows bio-inspired TENG-powered e-skin sensors for robotic tactile sensing applications. Characterization of the handshaking pressure and bending angles of each bionic finger during a human handshake proves the tactile sensis abilities of TENG e-skin sensors [83]. TENG e-skin sensors can be used to measure surface roughness and hardness [120]. Figure 9f shows a tiny tactile sensor based on dual-mode TENGs. This self-powered dual-mode sensor can interpret object contact and hardness by studying the contour of the current peak [121].

### 4.4. Smart Face Mask Based on Triboelectric Nanogenerators

**Figure 10:**
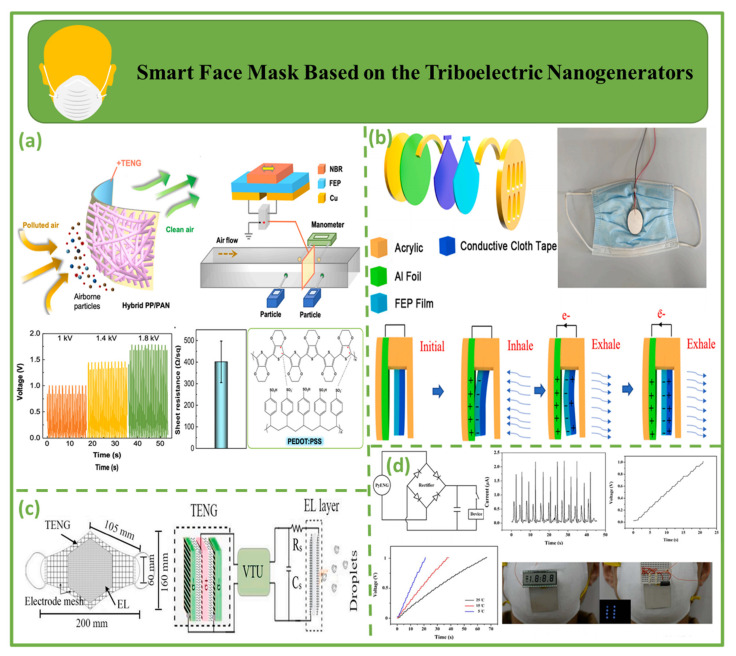
Smart face mask based on triboelectric nanogenerators. (**a**) The filtering principle of a tribocharge air filter face masks [122]. (**b**) Basic design of RS-TENG [123]. (**c**) Representation of the potential functionality of a triboelectric self-powered mask [124]. (**d**) Output efficiency produced by the PyNG when powered by human breathing [125].

Figure 10 depicts a triboelectric nanogenerator-based smart face mask, with the construction of a hybrid air filter mask shown in Figure 10a. A tribocharge-enhanced hybrid air filter mask with a 9.3–34.68 percent filtration improvement for particles of 0.3–2.5 µm compared to a state-of-the-art air filter utilized in disposable masks was designed for effective collection of nano- to micro-sized particulate matter. A modest pressure drop of approximately 110 Pa results in a significantly increased service life of 48 h with steady filtration effectiveness of 94 percent for 0.3–0.4 µm and 99 percent for 1–2.5 µm [122].

Figure 10b illustrate the principle of RS-TENG. A triboelectric nanogenerator for respiratory sensing (RS-TENG) was designed and connected to a facemask to provide respiratory monitoring capabilities. This design facilitated the development of multifunctional health monitoring devices during the COVID-19 pandemic due to its outstanding benefits, such as its compact size, ease of production, simple installation, and cost-effectiveness [123]. Figure 10c shows a triboelectric face mask. The recommended mask is layered, with the inner three layers working as a triboelectric (TE) filter and the outside layer serving as an electrocution layer (EL). Owing to the electric field between electrocution layers, virus particles are shocked in the EL. Four pairs of triboelectric textiles, PVC-nylon, PP-PU, latex rubber-PU, and PI-nylon, were investigated to assess mask efficacy [124]. Figure 10d shows the PyNG structure. PyNG can generate 42 V open-circuit and 2.5 μA short-circuit output signals. The maximum power at 50 MΩ was 8.31 μW. The remarkable performance and unfettered wearing mode of PyNG make it a suitable wearable energy harvester and self-powered multipurpose sensor [125].

### 4.5. Triboelectric Nanogenerators for Sleep Monitoring

**Figure 11:**
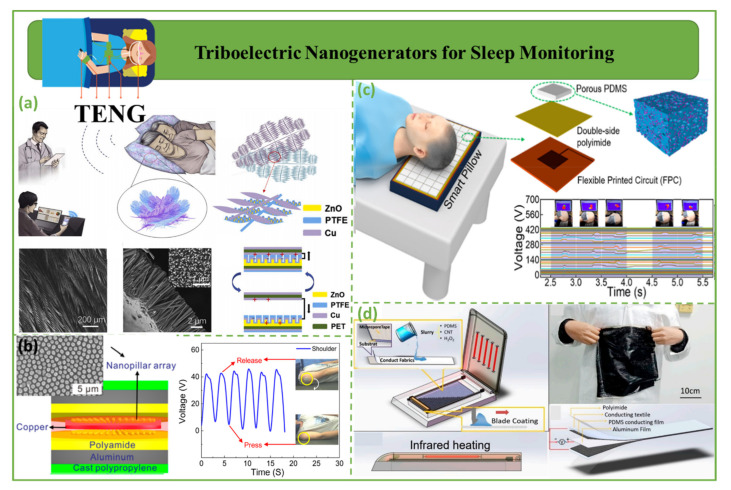
Triboelectric nanogenerators for sleep monitoring. (**a**) Structural design of a proposed smart pillow [127]. (**b**) The use of the TES to monitor the body during sleep [129]. (**c**) Utilization of an FB-TENG array in the form of a smart cushion for the purpose of monitoring head movement [130]. (**d**) Self-powered source for a bendable sensor for tracking human sleep [131].

Triboelectric nanogenerators for sleep monitoring are shown in Figure 11. Figure 11a shows a wireless sleep monitoring system for active healthcare and remote diagnostics. Inspired by pillow fillers, self-powered body-motion sensors with fractal down-like or feather-like structure were designed for daily bedding [126]. This technology may offer comfortable remote sleep healthcare and illness diagnostics for home-based sleep monitoring of the elderly, with the goal of reducing the risk of unexpected death during sleep [127]. Figure 11b shows a flexible and affordable triboelectric nanogenerator (TENG) based on a patterned aluminum–plastic sheet and an entrapped cantilever spring leaf for sleep–body movement monitoring [128]. This discovery may increase the usage of self-powered TENGs in healthcare and help build real-time mobile healthcare services [129]. Figure 11c demonstrates a sleep monitoring (FB-TENG) structure for a pressure-sensitive, non-invasive, and comfortable smart pillow that can monitor head movement in real time during sleep. The FB-TENG is made of pressure-sensitive and durable porous poly(dimethylsiloxane) (PDMS) with fluorinated ethylene propylene (FEP) powder. Kou et al. proposed a practical sensing device for sleep monitoring that could be expanded in the future to real-time monitoring of certain disorders, including brain ailments and cervical spondylosis [130]. Figure 11d shows a large-scale triboelectric nanogenerator with a flexible sleep sensor. This technology enables large-scale, low-cost TENG sensor production [131].

### 4.6. Self-Powered Nerve/Muscle Stimulation Based on Triboelectric Nanogenerators

**Figure 12:**
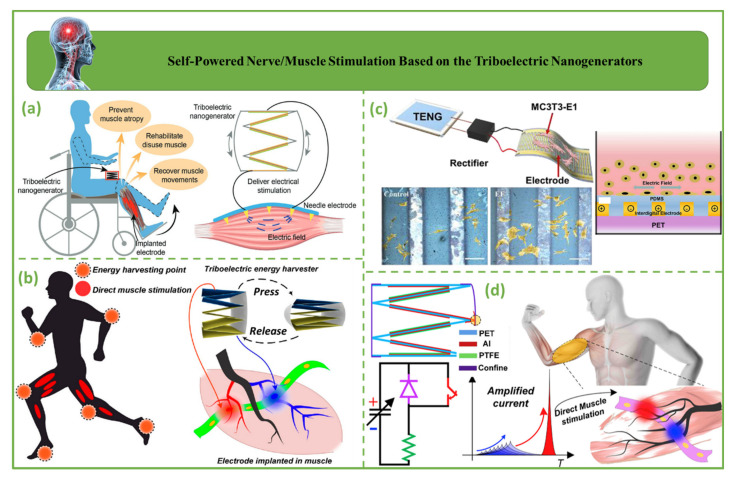
Self-powered nerve/muscle stimulation based on triboelectric nanogenerators. (**a**) Diagrammatic representation of electrical muscle activation directly driven by a TENG [133]. (**b**) Diagrammatic representation of electrical muscle stimulation directly driven by a TENG [38]. (**c**) Self-powered implantable electrical stimulator [25]. (**d**) D-TENGs are used for the purpose of direct muscle activation [39].

A self-powered nerve/muscle stimulation based on triboelectric nanogenerators is shown in Figure 12. Figure 12a demonstrates a self-powered system consisting of a stacked-layer triboelectric nanogenerator (TENG) and a multiple-channel epimysia electrode for direct stimulation of muscles. The two obstacles associated with direct TENG muscle stimulation were studied further in [38,39]. The optimal stimulation efficiency can be attained via systematic mapping using a multiple-channel epimysia electrode to address the first obstacle of enhancing the efficiency of low-current TENG stimulation [25,132]. The second difficulty is the stability of TENG stimulation. The force output produced by TENGs has been proven to be more stable than that of traditional square-wave and enveloped high-frequency stimulation [133]. Figure 12b depicts stacked-layer triboelectric nanogenerator (TENG)-driven electrical muscle stimulation using a flexible intramuscular electrode with several channels. This multiple-channel intramuscular electrode maps sparsely scattered motoneurons in muscle tissue, enabling high-efficiency TENG muscle stimulation despite the TENG’s moderate short-circuit current [38]. Figure 12c shows a self-powered implanted stimulator. Self-powered implanted electrical stimulators based on triboelectric nanogenerators (TENGs) could improve osteoblast adhesion, proliferation, and differentiation. Implanting a flexible TENG into living creatures verified the practicality of the self-powered electrical stimulator [25]. Figure 12d illustrates a diode-amplified triboelectric nanogenerator (D-TENGs). Diode-amplified triboelectric nanogenerators (D-TENGs) may improve direct muscle stimulation [39].

## 5. Applications, Challenges, and Future Trends of TENGs for Biomedical Sensors

**Figure 13:**
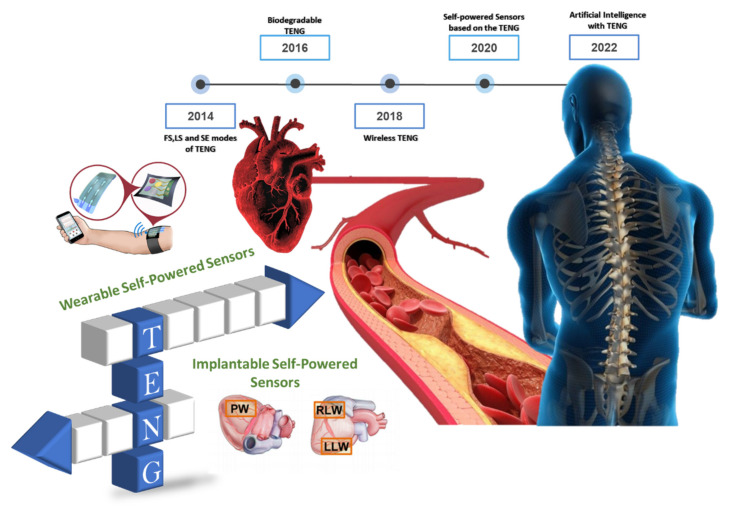
TENG biomedical sensor applications, limitations, and trends for the future.

Figure 13 illustrates challenges associated with TENGs and future tendencies. In January 2012, Wang’s group introduced the triboelectric nanogenerator concept, which employs contact electrification and electrostatic processes to harvest ambient mechanical energy [62,75,76,77,78,117,126,132,134,135,136,137,138]. TENGs now include biodegradable, wireless, blue energy, and smart city applications. When paired with AI, TENGs can power robots and self-powered sensors. In ubiquitous computing, TENGs are recommended for extension of sensor networks. Notwithstanding the papers outlining TENG-based devices for biomedical sensing, evaluating the performance of such devices can be difficult due to variances in working modes, testing, materials, and applications. It is incorrect to presume that one device performs better than another without considering other factors. Some suggestions for future research include:

1. Materials used in biomedical monitoring are anticipated to be flexible, light, stretchable, washable, attractive, skin-friendly, and even environmentally beneficial from the standpoint of wearability. As a result, researchers will gradually employ textile, rubber, hydrogel, shape-memory polymers, and other innovative functional materials to create well-designed TENG sensors.
2. With respect to sensing techniques, quantification could replace traditional two-stage judgment (i.e., “0” and “1”) on a transient pulse with no intermediate state, particularly for the control step. Furthermore, the composite mechanism of the intermediate state in a human-like intelligent sensor should be investigated further.
3. From the standpoint of technical integration, multiparameter systems can be built with advanced packaging and optimized modularization, and other novel technologies can be introduced to support the development of wearable biomedical monitors.

Table 1 shows a comparison of the performance output of various TENG devices for biomedical sensors, such as heart and respiration monitoring, blood pressure sensors, smart shoes, motion sensors [135], tactile sensors, smart face masks, sleep monitoring, and self-powered nerve/muscle stimulation. Most of the electrodes used in TENG are made of Al and Cu, most triboelectric layers comprising PDMS, PTFE, and Kapton.

## 6. Conclusions

For energy harvesting and sensing in healthcare and biological applications, triboelectric nanogenerators possess an abundance of advantageous characteristics, such as flexibility, low weight, and easy integration. Triboelectric nanogenerators confront a number of obstacles, such as fatigue, likely loss of elasticity, and damage to auxiliary components. Several features of TENG healthcare systems that provide wearable, minimally invasive, and uncomplicated solutions while gathering energy from human motion were investigated. Recent improvements in triboelectric devices were assessed in terms of their importance, structure, capabilities, performance, and future potential. The protective and therapeutic effects of TENG on numerous internal and exterior human organs need to be reported in detail. Finally, we discussed the growth of TENGs and the problems and possibilities they provide in healthcare and biological applications.

## Figures and Tables

**Table 1:** Summary of various TENG techniques for biomedical sensors.

Structure	Year	Authors	Applied Tribolayer	Electrode Type	Max Open-Circuit Voltage (V )	Max Short-Circuit Current	Current Density	Surface Power Density	Power Density and Power	Advantages/Disadvantages
Heart and respiration monitoring	2021	Shen et al. [81]	Kapton/PDMS	Cu	109	2.73 µA	-	-	-	Monitors the condition of the heart and respiration system/Incompatible with the cellular tissues of the heart in some cases
	2020	Li et al. [82]	PTFE	Cu	0.2–45	0.5–18 µA	-	0.6–15 W/m^2^	-	
	2019	Zhang et al. [83]	PTFE/nylon	Cu	40	-	-	-	-	
	2014	Zheng [87]	Kapton/PDMS	Al/Au	12	0.8 µA	-	8.44 mW/m^2^	-	
	2021	Zhao et al. [88]	Silicone rubber	Cu	3.67	51.74 nA	-	-	-	
	2016	Zheng et al. [89]	PTFE/Kapton/PDMS	Al/Cu	90	12 µA	-	107 mW/m^2^	-	
Blood pressure sensors	2019	Liu et al. [91]	PDMS/FEP	Cu	~70	-	-	-	-	Prevention of heart attack and stroke
	2016	Ma et al. [93]	Kapton/PDMS/PTFE	Al/Au	~15	4 µA	-	-	-	
Smart shoes	2017	Li et al. [94]	Dielectric elastomer	Conductive Elastomer	50	16.2 µA	~8 mA/m^2^	0.1 W/m^2^	-	Energy harvesting/increased vulnerability of shoes
	2013	Zhu et al. [95]	Kapton/PTFE	Al	220	600 µA	-	-	-	
	2013	Hou et al. [96]	PDMS/ITO	Cu/PET	220	40 µA	~0.08 mA/cm^2^	-	1.4 mW	
	2018	Jao et al. [97]	PTFE	Metal/chitosan-glycerol	130	15 µA	10 mA/m^2^	-	-	
	2018	Chen et al. [98]	PGS/CNTs	Salt	170	11 µA	200 mA/m^2^	-	185.2 µW	
	2021	Yun et al. [99]	FEP	Al	3 k	20 µA	-	-	3 mW	
Motion sensors	2022	Matin Nazar et al. [100]	Kapton	Al/Cu	21.9	-	-	-	70 µW	Monitors walking behavior and helps to improve the treatment process/exposed sensors subject to increased vulnerability
	2022	Zeng et al. [101]	FEP/silicone/PTFE/nylon	Carbon black/Al	468	10.4 µA	-	-	1.25 mW	
	2018	Tian et al. [102]	Silicone	Ni/conductive silicone	380	11 µA	-	-	1.638 mW	
	2021	Zhang et al. [103]	PTFE/cotton	Conductive cotton	556	26 µA	-	0.66 mW/cm^2^	-	
	2019	Gogurla et al. [104]	PDMS/silicone	AgNWs/Al/Cu/PET	110	~0.1 µA	-	2 mW/m^2^	-	
	2020	He et al. [105]	MXene/PDMS	Cu–Ni/textile	225	-	30 µA/cm^2^	10 mW/cm^2^	-	
Tactile sensors	2020	Zhou et al. [115]	TPU mats	AgNWs/rGO	202.4	-	-	6 mW/m^2^	-	Increase efficiency, energy harvesting and aids in the diagnosis of disease/challenges associated with washing; can cause skin sensitivity and discomfort in some users
	2019	Zhao et al. [116]	PDMS	PAMPS ionogel	3.3	2.3 nA	-	-	-	
	2016	Wang et al. [78]	PDMS/Kapton	PET	~60	-	-	-	-	
	2021	Wu et al. [119]	PDMS	AgNWs	78.7	26.5 µA	-	33.75 W/m^2^	-	
	2019	Yao et al. [120]	PDMS	AgNWs	3.48	26.29 nA	-	-	-	
	2017	Li et al. [121]	PDMS	AgNWs/CuNWs/Al	90	9 µA	-	-	-	
Smart face masks	2021	Wang et al. [122]	FEP/NBR	Cu/AgNW	1.8 k	-	-	-	-	Increases performance and efficiency/can cause skin sensitivity in some users
	2022	Lu et al. [123]	FEP/acrylic	Al	8	0.8 µA	-	-	-	
	2021	Ghatak et al. [124]	PVC/PP/latex rubber/PI	Nylon/Pu	~90	~25 mA	-	-	400 mW	
	2017	Xue et al. [125]	PVDF	Al	42	2.5 µA	-	-	8.31 µW	
Sleep monitoring	2020	Zhang et al. [127]	PTFE	Cu	~350	~40 µA	-	-	11.6 mW	Improves the treatment of insomnia and sleep disorders
	2016	Song et al. [129]	CPP/PA	Al/Cu	55	0.9 µA	-	~120 mW/m^2^	-	
	2022	Kou et al. [130]	PDMS/Kapton	Al	~65	~0.7 µA	-	-	-	
	2018	Ding et al. [131]	PDMS	Al/textile electrode	~16	-	-	-	-	
Self-powered nerve/muscle stimulation	2019	Wang et al. [133]	PTFE	Al	-	55 µA	-	-	-	Monitoring the condition of the nerve/muscle stimulation system/incompatible with the cellular tissues in some people
	2019	Wang et al. [38]	PTFE	Al	47	35 µA	-	-	95 µW	
	2019	Tian et al. [25]	PTFE/PDMS	Au/PET	100	1.6 µA	-	-	-	
	2019	Wang et al. [39]	PTFE	Al	-	40 µA	-	-	~500 µW	

## Data Availability

The data presented in this study are available upon request from the corresponding author.
